# Supplementary material for: Cancer metastasis is related to normal tissue stemness
Source: PLoS One. 2022 Nov 22;17(11):e0277811. doi: 10.1371/journal.pone.0277811 (PMC9681098; doi:10.1371/journal.pone.0277811)
Supplement: Attachment — Submitted filename: Response to Reviewers.pdf [file pone.0277811.s002.pdf]

Dear Reviewers,

We are pleased to submit our revised manuscript (R2) entitled "*Cancer metastasis is related to normal tissue stemness*".

We have carefully revised it based on editor comments and all your comments. Following all questions **(Q1-Q4)**, we made revisions with feedback on all questions **(A1-A4)**. We hope that this revised manuscript meets the requirements for publication.

We are very grateful to the editors and your valuable comments and suggestions.

Thank you and best regards.

Sincerely yours,
Corresponding Author

PS: -----------Questions (Q1—Q4) and answers(A1—A4)----------------------

[Q1]: abstract line 8: "We data-mine this data mining.." delete "mining" **[Editor comments]**
[A1]: This word has been deleted (Abstract line 8).

[Q2]: last paragraph of the introduction: the statement "to conclude clinical cancer metastasis data and stem cell index data" -is not clear. Do the authors mean "to integrate ..." or better "to derive insights on when metastasis begins, based on joint mining of both data sets"? **[Editor comments]**
[A2]: We revised to:

This paper attempts to derive insights on when metastasis begins based on joint mining of the clinical cancer metastasis data sets with stem cell index [24] data sets representing the stem cell content of normal or tumor tissues. (Line 34 … Line 35)

[Q3]: Reviewer #2: The author responded adequately to the requests of the previous reviewers making the paper more interesting for a publication on PLOS One. Within the discussion, the author is asked to clarify the efficiency and effectiveness of what has been identified in clinical practice. **[Reviewer #2]**
[A3]: We further supplement the following discussion:

NS is also an objective and numerical quick biochemical index with high clarity and criteria (e.g., NS>0.5). With NS's fast, safe, and convenient standards, clinicians can find patients

with an increased risk of tumor metastasis. Therefore, the NS index has high operability and is a promising clinical application. (Line 240)

[Q4]: It remains in the opinion of the undersigned that the clinical application of what has been identified requires greater verification in daily practice. **[Reviewer #2]**

[A4]: We strongly agree with the reviewer's point of view. We supplement the discussion below.

The clinical application of NS requires more significant verification in daily practice. At present, the data of clinical research on NS is minimal, and we need an extensive NS detection database to judge the NS's effectiveness. (Line 247)
